# Mapping the Dynamics of Inhibitors and Facilitators of Exercise Behavior Within the Transtheoretical Model: Nationwide Cross-Sectional Study Using Text Mining Analysis

**DOI:** 10.2196/77400

**Published:** 2025-10-24

**Authors:** Kosuke Sakai, Kota Fukai, Yuko Furuya, Shoko Nakazawa, Kei Sano, Masayuki Tatemichi

**Affiliations:** 1 Department of Preventive Medicine, Tokai University School of Medicine, 143 Shimokasuya, Isehara, Kanagawa, 2591193, Japan, 81 463 93 1121 ext 2612, 81 463 92 3549; 2 Department of Ophthalmology, Jikei University School of Medicine, Tokyo, Japan

**Keywords:** transtheoretical model, text mining, exercise, inhibitor, facilitator

## Abstract

**Background:**

The transtheoretical model (TTM) explains behavior changes through sequential stages influenced by the balance of perceived benefits and barriers. Although previous studies have identified the inhibitors and facilitators of exercise behavior, only a few have elucidated how these factors vary across the stages of behavior change.

**Objective:**

This study aimed to identify the inhibitors and facilitators of each stage of behavior change using text mining.

**Methods:**

A nationwide cross-sectional study was conducted using an internet-based questionnaire with a panel of approximately 2 million members. From this panel, 93,460 individuals were randomly selected and invited to participate via email and app push notifications. A total of 1500 valid responses were included in the analysis through stratified sampling based on sex, age group, and geographic region. The participants self-assessed their stages of change. Two open-ended questions captured the perceptions of inhibitors and facilitators of exercise behavior. Text responses were analyzed in a 4-step process: morphological analysis to extract frequently used words, correspondence analysis to visualize relationships between frequently used words and the 5 change stages, conceptual categorization with coding rules, and creation of heat maps to illustrate stage-specific categories in inhibitors and facilitators.

**Results:**

Out of 1500 respondents, 754 (50.3%) were males and 355 (23.7%) individuals were in the 50‐59 age group. Stage percentages were precontemplation 24.3% (365/1500), contemplation 23.5% (352/1500), preparation 21.3% (320/1500), action 5% (75/1500), and maintenance 25.9% (388/1500). The inhibitors and facilitators were described using 9893 words and 8372 words, respectively. Inhibitors clustered into 7 categories; most frequent were time (408/1500, 27.2%), motivation (253/1500, 16.9%), health (189/1500, 12.6%), and working (158/1500, 10.5%). Facilitators formed 8 categories; most frequent were subjectivity (155/1500, 10.3%), relationship (93/1500, 6.2%), opportunity (84/1500, 5.6%), reward (78/1500, 5.2%), and record (78/1500, 5.2%). Stage-specific patterns emerged: inhibitors shifted from motivation and health (precontemplation) to family, time, and working (contemplation and preparation), opportunity (action), and weather and health (maintenance). Facilitators of reward, health, and record rose progressively from precontemplation to maintenance.

**Conclusions:**

This study enhances our understanding of the dynamic mechanisms underlying exercise behavior change by identifying how specific inhibitors and facilitators vary across behavioral stages. The findings underscore the need to tailor interventions based on individuals’ readiness to change, rather than relying on one-size-fits-all strategies. For both practitioners and policymakers, incorporating behavioral stage frameworks into assessments and interventions, such as those conducted during health checkups or workplace programs, may improve the effectiveness and sustainability of physical activity promotion.

## Introduction

Exercise habits can improve mental and cardiovascular health [1,2]. A key health policy guideline for Japan, “Health Japan 21,” placed a high priority on exercise habits, with the goal of increasing the percentage of people with exercise habits from 28.7% in 2019 to 40% by 2032 [3]. In this guideline, “exercise habits” are defined as engaging in exercise for at least 30 minutes per session, at least twice a week, and continuing for at least 1 year. Despite this policy target, the exercise habit rate has decreased to 27.3% in 2023 due to the coronavirus disease 2019 epidemic [4]. The focus is on providing effective interventions to promote exercise habits.

The behavior change process is explained using the transtheoretical model (TTM) [5,6]. The model categorizes the process of behavior change into 5 stages—precontemplation, contemplation, preparation, action, and maintenance—assigns individuals to one of these stages, and conceptualizes each stage as dynamically evolving over time. Various factors are associated with the acquisition of exercise habits, including individual characteristics, societal norms, culture, and the environment [7]. In the TTM, the psychologically influential factors are categorized into cognitive process of change, intrinsic mental shifts, and behavioral process of change, which are influenced by environmental and external support [8]. In addition, a person’s stage can change depending on the balance between perceived benefits and barriers to exercise. Interventions designed using the TTM have been shown to be highly effective in changing behaviors [9]. Unlike other models that focus solely on intention or beliefs, such as the health belief model or the theory of planned behavior, the TTM is uniquely suited to capture temporal changes and stage-specific factors. This makes it particularly appropriate for identifying which facilitators and inhibitors are most relevant at each stage of exercise behavior. Furthermore, in Japan, the TTM has been applied in public health efforts such as the implementation of “tokutei-kenko-shinsa” (specific health checkups), a nationwide preventive health screening program targeting metabolic syndrome. Thus, the TTM has both theoretical and practical relevance for understanding and promoting exercise behavior in the Japanese population [10].

Factors influencing behavior change can be divided into inhibitors and facilitators [11]. Inhibitors include diseases, psychological barriers, time constraints, and poor access to exercise environments. Facilitators include self-efficacy, positive feelings toward exercise, good health, and social support [11]. The extent to which these factors influence exercise behavior varies depending on the individual stages of behavior change [12]. A previous study has found that physical, mental, and environmental barriers have a significant influence on the precontemplation and contemplation stages, perceived behavioral control is significant in the contemplation stage, and self-efficacy and social support play a significant role in the action and maintenance stages [13]. The relationship between these factors and the stages of behavior change is complex [14].

Recently, text mining has been recognized as an effective method for elucidating increasingly complex phenomena [15,16]. Previous studies aiming to identify inhibitors and facilitators of exercise habit have collected qualitative data through semistructured interviews and focus group discussions, which were then analyzed and interpreted by researchers to identify and categorize key concepts [11,13,17,18]. Text mining enables the visualization and quantitative evaluation of relationships between words in free-text responses and respondents’ demographic characteristics. Text mining has been used in various public health studies, leveraging its high reproducibility and capacity to process large volumes of data [19,20]. Studies using text mining to analyze the influential factors in the exercise habits have been limited to college students and workers in specific occupations [21,22]. To the best of our knowledge, no previous studies have used this method to analyze the relationship between behavior change stages and both inhibitors and facilitators. Text mining may yield valuable insights into the inhibitors and facilitators, as well as elucidate the complex relationships between these factors and their respective stages.

This study aimed to identify the inhibitors and facilitators of each stage of behavior change using text mining. Unraveling the complexity of the factors influencing behavior change will deepen our understanding of exercise habit formation and support the development of individualized interventions.

## Methods

### Study Design and Participants

This was a nationwide cross-sectional study in Japan using an internet questionnaire survey, which was administered in November 2024. The survey was conducted by Rakuten Insight Inc, a web-based research firm in Tokyo, Japan. It was one of the largest and most widely used web-based research providers in the country, with a panel of approximately 2 million members. The company has also been used in previous peer-reviewed public health research [23,24]. This study was reported in accordance with the STROBE (Strengthening the Reporting of Observational Studies in Epidemiology) guidelines for cross-sectional observational studies (Checklist 1) and the CHERRIES (Checklist for Reporting Results of Internet E-Surveys) checklist for Internet-based surveys (Checklist 2) [25,26].

The participants were recruited from the company’s registry. The inclusion criterion was aged 20‐69 years living in Japan. To ensure a representative sample, stratified sampling was used across 200 segments based on sex (male and female), 5-year strata age groups, and 10 geographical regions (Hokkaido, Tohoku, North Kanto, South Kanto, Hokuriku, Tokai, Kinki, Chugoku, Shikoku, and Kyushu) in accordance with the distribution of population estimates from the 2020 census [27]. Surveys were conducted until 1500 valid responses were obtained, with target sample quotas established for each segment. The sample size was determined pragmatically as the maximum number of responses that could be obtained within the available budget. The questionnaire consisted of one question per page, totaling 21 pages. Respondents were able to review and change their answers before final submission.

**Figure 1.**
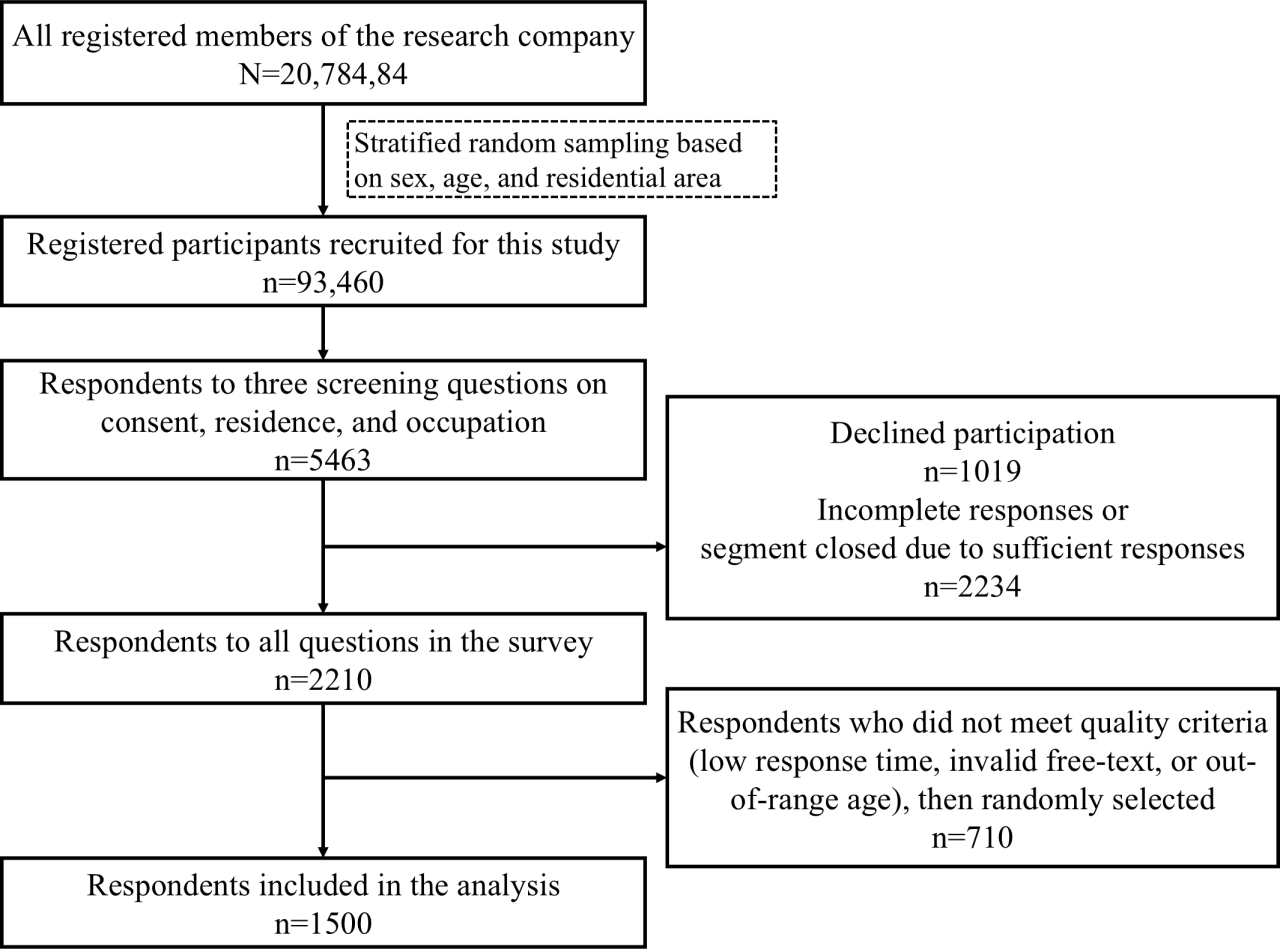
Flowchart of participant selection for analysis.

Figure 1 illustrates the process of selecting participants for the final analysis. From a pool of 2,078,484 registered members of the survey panel, 93,460 participants were invited to take part in the study via email and push notifications through the company’s app. These participants were selected using stratified random sampling based on sex, age, and residential area. Of these, 5463 individuals responded to 3 initial screening questions regarding their consent to participate, place of residence, and occupation. A total of 3253 individuals were excluded at this stage due to declining participation, providing incomplete responses, or belonging to quota-saturated demographic segments. Among the 2210 individuals who responded to the survey, 1500 were randomly selected for analysis using the following procedure. First, a validity flag was assigned to samples that met predefined criteria. Before flagging, respondents were excluded if they (1) had response times below a threshold (defined as less than the median response time multiplied by 0.3 for PC users or 0.2 for smartphone users), (2) provided clearly inappropriate answers in free-text fields, or (3) reported an age outside the target range (18‐79 y). Next, a random number was assigned to each valid sample using the RAND function in Excel, which generates a random number between 0 and 1. The samples were then sorted in ascending order based on the random number, and the top 1500 were selected for inclusion in the final dataset. Cookies and IP addresses were not used in this survey. This was a closed survey conducted through a commercial Internet survey company. Respondents were required to log in with their unique panel ID before accessing the questionnaire. Once a panelist had completed the survey, the questionnaire was no longer displayed upon subsequent logins, thereby preventing duplicate entries.

### Assessment of Behavior Change Stage in the Transtheoretical Model

Behavior change stage was measured using the Japanese version of the Stages of Exercise Behavior Change Scale, which has been validated in Japanese populations and shown to have acceptable reliability and construct validity [28]. This required respondents to select the appropriate item from the following 5 options: precontemplation, “I currently do not exercise and do not intend to exercise for the next 6 months;” contemplation, “I currently do not exercise, but I intend to exercise within the next 6 months;” preparation, “I currently exercise but not regularly;” action, “I currently exercise regularly, but I have only begun to do so within the past 6 months;” and maintenance, “I currently exercise regularly and have been doing so for >6 months.” Regular exercise was defined as exercise lasting >20‐30 min per set, 2‐3 times per week.

### Assessment of Inhibitors and Facilitators for Exercise

We collected free responses regarding the inhibitors and facilitators of exercise using 2 open-ended questions. To elicit open-ended responses, participants were presented with the following prompts: (1) For inhibitors: “What kinds of obstacles do you face in continuing your exercise habits? Please describe them in as much detail as possible.” (2) For facilitators: “What kinds of things have helped or supported you in continuing your exercise habits? Please describe them in as much detail as possible.”

Participants were instructed to list as many ideas as they could think of and were encouraged to be specific. These questions were mandatory, and respondents could not proceed to the next section without entering a response.

### Assessment of Respondents’ Characteristics

The demographic information of the respondents, including sex (male and female), age (20‐69 y) at the date of response as identified by date of birth, marital status (married, never married, and divorced or deceased), education (junior or high school, vocational school or junior college, university, graduate school, currently enrolled, and other), and household income (<2.0, 2.0‐3.9, 4.0‐5.9, 6.0‐7.9, 8.0‐9.9, 10.0‐13. 9, and ≥14.0 million Japanese yen; US $1 = ¥150, based on the average exchange rate in November 2024), was collected.

### Statistical Analyses

The text mining analyses were performed in 4 steps. Statistical analysis was performed using KH-coder (version 3.02c official package, SCREEN Advanced System Solutions Co, Ltd) [29]. KH Coder was initially developed as an open-source software for academic research and gained wide recognition due to its accessibility and functionality. Over time, its analytical methods were validated through numerous peer-reviewed publications across fields such as public health and social sciences [16,19,20,30]. Its core methods—such as morphological analysis, co-occurrence networks, and correspondence analysis—have been repeatedly used to analyze publicly available text data, thereby ensuring transparency and reproducibility. The consistency of results reported across independent studies provides evidence supporting the methodological validity and reliability of this software. We analyzed the descriptions in Japanese and translated the words into English.

### Morphological Analysis

In the first step, we performed a morphological analysis to determine frequently used words, their parts of speech, and the number of occurrences using the text-mining dictionary ChaSen Morphological Analyzer, which was the default engine in KH Coder and has been widely selected for its compatibility and proven utility in academic research settings [16,20].

### Correspondence Analysis

In the second step, we performed a correspondence analysis to visualize the relationship between the top 40 most frequent words and the 5 stages of change, stratified by inhibitors and facilitators. Correspondence analysis was classified as a multivariate analysis and selected when the variables were categorical data. This method aids in visually understanding the relationships among categorical data, which can be represented using a 2D scatterplot [31]. Categories located farther from the origin and closer to the direction of self-rated health levels were considered more specific. Correspondence analysis is often used in marketing to visualize the associations between multiple categorical variables and consumer characteristics. In the field of public health, we have applied this method in our previous research to identify key personnel responsible for employee health management in the workplace and to determine appropriate marketing strategies through various media channels [30].

### Conceptualization and Coding Rules

In the third step, we conceptualized the descriptions into several categories from the frequent word lists of inhibitors and facilitators. The coding rules were developed through an iterative process by the first author (KS) who has published multiple peer-reviewed studies using text mining, in collaboration with KF. To ensure the contextual validity of the coding rules, all free-text responses were read in full before conducting morphological analysis. After extracting the frequently used words through morphological analysis, 2 researchers independently reviewed all responses containing those words to understand the specific contexts in which each word was used. Similar words were grouped together, and each group was assigned a conceptual label that reflected the underlying theme. After drafting the initial coding rules, the researchers revisited all responses in which those words appeared to confirm the appropriateness of the assigned categories. Words that were used in multiple or ambiguous contexts were excluded from the coding rules to preserve specificity. After a 1-month interval, the researchers repeated the process to refine and finalize the coding scheme, thereby improving consistency and minimizing potential bias. These processes were carried out through discussions between the 2 researchers. In cases where disagreements arose during the categorization process, the supervising author (MT) was consulted to provide a third opinion and reach consensus. Coding rules were established for each category based on words that appeared at least 10 times. We excluded stop words, such as conjunctions and prepositions and words used in a variety of contexts, such as Nothing, Not, and Exercise. We calculated the number of responses for each category based on coding rules.

### Heat Maps

In the fourth step, we created heat maps to visualize how each category varied across the stages of change. The heat maps visualized the variation in the mentions of each category using Pearson’s residual scales. Percentages were calculated using the number of responses in each stage as the denominator and the number of responses mentioning each category as the numerator. Pearson residuals indicate the degree to which observed values deviate from expected values in the analysis of mentions in a particular category. The heat map displays the color intensity when mentions of a particular category occur more or less frequently than expected.

Pearson residual=(O−E)/√E

O: observational number of responses mentioning the category.

E: expected number of responses mentioning the category.

The process of developing coding rules and visualizing conceptual categories through heat maps has been effectively applied in our previous studies, such as an investigation into the supportive behaviors of supervisors with high work engagement and an analysis of trends in occupational health research over the past three decades [16,19]. These applications have demonstrated the utility and validity of the approach.

### Sensitivity Analysis

To assess the robustness of the findings, we conducted sensitivity analyses stratified by sex (males and females) and education (less than university and university or more). The same coding rules and analytical procedures described above were applied separately to each group to examine whether the stage-specific distribution patterns of inhibitors and facilitators were consistent.

### Ethical Considerations

This study was approved by the Research Ethics Committee of the Tokai University School of Medicine (24R109). Informed consent was obtained from all the respondents before their participation in the questionnaire survey. To incentivize participation, respondents who passed the screening questions received 2 points, and those who completed the full survey received from 15 to 17 points. These points were equivalent to 1 yen per point and could be exchanged for merchandise on an affiliated online platform. All responses were anonymized by the survey company before being provided to the researchers, and no personally identifiable information was collected.

## Results

### Characteristics of Respondents

Respondents’ characteristics are listed in Table 1. Of 1500 respondents, 754 (50.3%) were males. In addition, 23.6% (178/754) males and 23.7% (177/746) females were in their 50 s. A total of 50.8% (383/754) males and 56.7% (423/746) females were married. Household incomes between 4 million and 5.99 million yen (US $1 = ¥150) were reported by 30.1% (227/754) men and 23.5% (175/746) women. In terms of the stage of change, 29.6% (223/754) men were in maintenance, 23.9% (180/754) in preparation, and 22.5% (170/754) in precontemplation; 28.6% (213/746) women were in contemplation, 26.1% (195/746) in precontemplation, and 22.1% (165/746) in maintenance. Because all questionnaire items were mandatory, there were no missing values among the analyzed participants.

**Table 1.** Characteristics of respondents.

	Males, n (%)	Females, n (%)
Total	754 (100)	746 (100)
Age group (years)		
20‐29	129 (17.1)	122 (16.4)
30‐39	135 (17.9)	131 (17.6)
40‐49	170 (22.5)	165 (22.1)
50‐59	178 (23.6)	177 (23.7)
60‐69	142 (18.8)	151 (20.2)
Marital history		
Married	383 (50.8)	423 (56.7)
Divorce or bereavement	42 (5.6)	85 (11.4)
Unmarried	329 (43.6)	238 (31.9)
Education		
Junior, high school	206 (27.3)	208 (27.9)
Vocational school, junior college	105 (13.9)	268 (35.9)
University	364 (48.3)	249 (33.4)
Graduate school	57 (7.6)	9 (1.2)
Currently enrolled	18 (2.4)	11 (1.5)
Other	4 (0.5)	1 (0.1)
Household income, million yen^a^		
<2.0	75 (9.9)	109 (14.6)
2.0 to 3.9	135 (17.9)	181 (24.3)
4.0 to 5.9	227 (30.1)	175 (23.5)
6.0 to 7.9	128 (17)	116 (15.5)
8.0 to 9.9	82 (10.9)	78 (10.5)
10.0 to 13.9	69 (9.2)	54 (7.2)
≥14.0	38 (5)	33 (4.4)
Stage of change		
Precontemplation	170 (22.5)	195 (26.1)
Contemplation	139 (18.4)	213 (28.6)
Preparation	180 (23.9)	140 (18.8)
Action	42 (5.6)	33 (4.4)
Maintenance	223 (29.6)	165 (22.1)

a 150 Japanese yen = US $1.

### Morphological Analysis

In the first step, we detected inhibitors using 9893 extracted words and 1157 unique words and facilitators using 8372 extracted words and 1241 unique words. Frequent words described for inhibitors, those appearing at least 10 times, are shown in Multimedia Appendix 1, and frequent words described for facilitators are shown in Multimedia Appendix 2. The inhibitors included time (N=387), job (N=154), exhausted (N=58), eagerness (n=49), troublesome (n=46), and busy (n=44). The facilitators included nothing (n=542), gymnasium (n=56), time (n=52), together (n=48), understand (n=44), application (n=38), and goal (n=32*).*

### Correspondence Analyses

**Figure 2.**
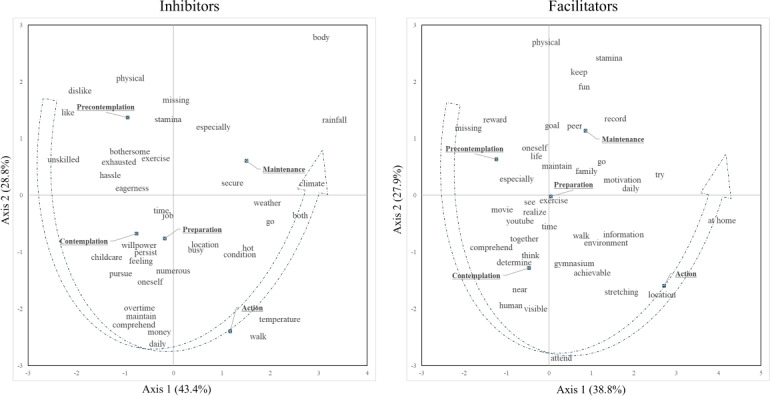
This figure presents the results of the correspondence analysis, illustrating the relationship between the stages of behavioral change and frequently mentioned words related to inhibitors (left panel) and facilitators (right panel) of exercise habits.

In the second step, Figure 2 presents the results of the correspondence analyses to visualize the relationship between the stages of change and the 40 specific words in the inhibitors and facilitators. The stages of change were arranged counterclockwise for both the inhibitors and facilitators. A spectrum was drawn from the appearance of the word characteristics at each stage. In the inhibitors, dislike, like, bothersome, and unskilled appeared in the precontemplation period; childcare, willpower, overtime, and money appeared in the contemplation and preparation; walk and temperature appeared in the action period; and body, rainfall, and climate appeared in the maintenance period. In the facilitators, missing and reward emerged in precontemplation; near, human, and visible emerged in contemplation; location and stretching emerged in action; and stamina, fun, record, and peer emerged in maintenance.

### Conceptualization and Coding Rules

In the third step, the inhibitors and facilitators were structured into several categories using coding rules, as shown in Table 2. The inhibitors were divided into seven categories (motivation, health, weather, family, time, opportunity, and working). In response to the inhibitors, 408 (27.2%) respondents referred to the category of time, including words, such as time, busy, and capacity*.* Similarly, 253 (16.9%) respondents mentioned motivation, 189 (12.6%) mentioned health, and 158 (10.5%) mentioned work. A total of 572 (38.1%) participants did not use any of the words included in the coding rules. After reading all the responses in the category of no coding, we observed that 255 respondents answered, “I do not know” or “nothing in particular.” The facilitators comprised 8 categories (reward, subjectivity, opportunity, relationship, health, record, digital device, and time). Among the facilitators’ responses, 155 (10.3%) referred to the category of subjectivity, which included words such as understand, achievable, comprehend, and determine. Similarly, 93 (6%) and 84 (6%) participants mentioned the relationship and opportunity categories, respectively. There were 956 (63.7%) responses not referring to any categories (no coding), and 617 of these responses had no meaning, such as “I do not know” or “nothing in particular.”

**Table 2.** Conceptualization, coding rules, and reference conditions on inhibitors and facilitators.

Factors and categories		Words in coding rules	Reference counts and rate, n (%)
Inhibitors			
	Motivation	eagerness, troublesome, hassle, motivation, feeling, bothersome, willpower, like, dislike, unskilled	253 (16.9)
	Health	exhausted, stamina, condition, physical, knee, fatigue, backache, body	189 (12.6)
	Weather	weather, climate, rainfall, hot, chilly, temperature	98 (6.5)
	Family	chores, parenting, caregiving, childcare	71 (4.7)
	Time	time, busy, capacity	408 (27.2)
	Opportunity	gymnasium, location, money	48 (3.2)
	Working	job, overtime	158 (10.5)
	No coding^a^		572 (38.1)
Facilitators			
	Reward	point, motivation, fun, effect, reward, capable	78 (5.2)
	Subjectivity	understand, achievable, comprehend, determine	155 (10.3)
	Opportunity	gymnasium, location, environment, institution	84 (5.6)
	Relationship	together, peer, human, family	93 (6.2)
	Health	health, stamina, body, physical	68 (4.5)
	Record	goal, weight, record, strides	78 (5.2)
	Digital device	application, YouTube, movie	70 (4.7)
	Time	time, job	68 (4.5)
	No coding^a^		956 (63.7)

a No coding: responses that did not use any of the words listed in the coding rules above.

### Relationship Between the Stages of Change and the Categories of Inhibitors and Facilitators

**Figure 3.**
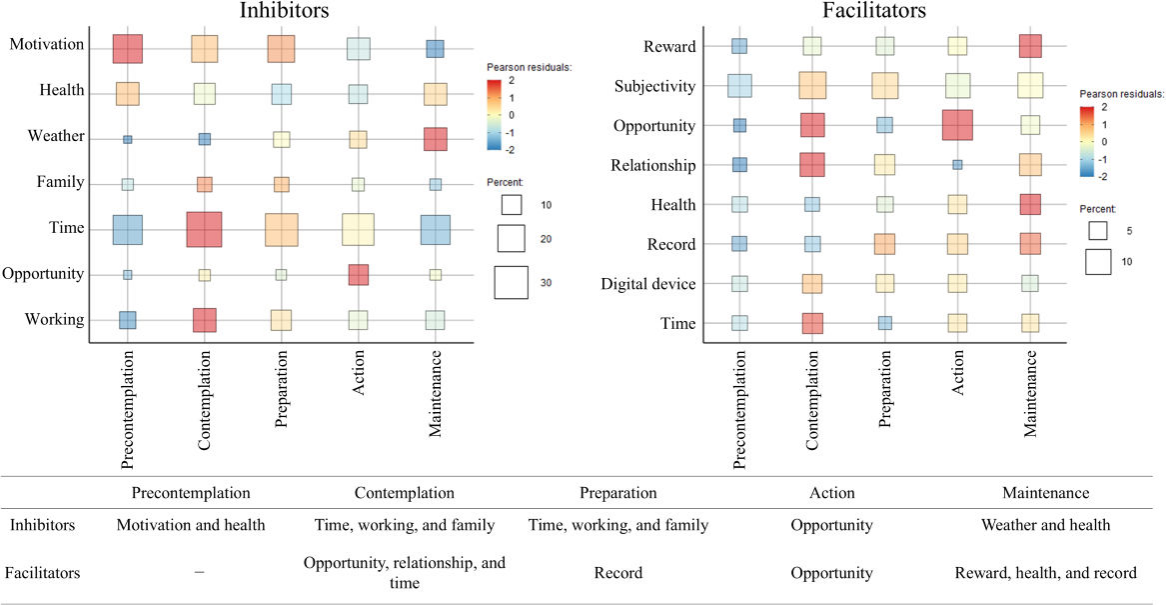
This heat map illustrates the relationship between the stages of behavioral change and the categories of inhibitors (left) and facilitators (right).

In the fourth step, the heat map in Figure 3 shows the association between the stages of change and the categories of inhibitors and facilitators. The color indicates the bias of the distribution in each category in the horizontal direction, and the size of the square indicates the percentage of mentions at each stage in the vertical direction. In terms of inhibitors, motivation decreased and weather increased as the behavior change stage progressed. The categories of family, time, and work were mentioned in contemplation and preparation, whereas opportunity was mentioned in action. Among the facilitators, reward, health, and record increased as the behavior change stage progressed. Opportunity was mentioned in contemplation and action, whereas relationship and time were mentioned in contemplation. The numbers and percentages of mentions of the categories by stage are detailed in Multimedia Appendix 3.

The results of the sensitivity analysis stratified by sex and education are presented in Multimedia Appendix 4 (inhibitors by sex), Multimedia Appendix 5 (facilitators by sex), Multimedia Appendix 6 (inhibitors by education), and Multimedia Appendix 7 (facilitators by education). For inhibitors, similar trends in category–stage associations were observed, even when the data were stratified by sex and educational attainment. For facilitators, the frequency of mentions across all categories remained consistently low in the precontemplation stage across all sensitivity analyses. However, beyond the precontemplation stage, the associations between facilitator categories and stages of change varied by sex and education, and no consistent patterns were observed.

## Discussion

### Principal Findings

In this study, a text-mining analysis of open-ended responses about the inhibitors and facilitators of exercise behavior revealed a complex relationship between the stages of change and these factors. Inhibitors were structured into 7 categories (motivation, health, weather, family, time, opportunity, and working) and facilitators into 8 categories (reward, subjectivity, opportunity, relationship, health, record, digital device, and time). The inhibitors included motivation and health in the precontemplation; time, working, and family in the contemplation and preparation; opportunity in the action; and weather and health in the maintenance. As precontemplation progressed to action, reward, health, and record emerged as facilitators. The findings of this study will contribute to a better understanding of the complex mechanisms underlying exercise habit formation and will inform the development of personalized interventions.

The following inhibitors changed as the behavior change stage progressed: motivation, health, flexibility (time, working, and family), opportunity, and weather. Overcoming these inhibitors makes it possible to acquire exercise habits. Similar inhibitors have been reported previously. A systematic review of the determinants of physical activity among adults in the United Kingdom identified lack of time, health problems, and language barriers as inhibitors [32]. A study of individuals with musculoskeletal disorders identified health conditions, time constraints, psychological barriers, and limited access to opportunities, including weather, as inhibitors [11]. To support the acquisition of exercise habits, these inhibitors need to be overcome. In a systematic review and meta-analysis, interventions involving motivational interviewing have been reported to be effective in promoting physical activity and exercise and reducing sedentary time [33]. Motivational interviewing of patients with cancer has reported effects on the acquisition of exercise habits over a period of ≥6 months [34]. Continuous sessions with a physiotherapist and motivational interventions are effective in improving exercise adherence in older adults with chronic low back pain to improve exercise adherence [35]. However, interventions using digital devices that have emerged in recent years have not been proven effective in promoting exercise in patients with musculoskeletal pain [36]. These results indicate the need for ongoing support from specialists. Time management and opportunities such as work–life balance, access to gyms, and financial resources can contribute to the development of exercise habits only when individuals are in good health and sufficiently motivated to exercise.

In the correspondence analyses, the facilitators showed no more fluid relationships with the behavior change stage than the inhibitors. In addition, the results of sensitivity analysis stratified by sex and education for the relationship between facilitators and stages were inconsistent. One possible reason for this is that facilitators vary according to the individuals’ backgrounds. In a qualitative study of individuals with chronic back pain, professional supervision and group practice served as facilitators, because pain was the primary inhibitor [18]. For those with depressive symptoms, emotions, beliefs about capability, and intentions were barriers; therefore, improving the emotional domain was a facilitator [37]. The facilitators depended on the inhibitors possessed by individuals.

As the behavior change stage progressed, reward, health, and record emerged as facilitators. Recording this process is expected to facilitate the observation of changes in the acquisition of exercise habits. As individuals build stamina, notice physical changes, and experience positive emotional rewards, they may progress to the maintenance stage. Setting goals and tracking weight and step counts enhance intervention effectiveness [38]. A cycle was observed in which self-efficacy increases as individuals progress through the behavior change stages, further boosting their motivation to exercise [38]. Tracking progress may act as a trigger for this cycle.

Facilitators were more frequently described in relation to behavioral processes that involve specific actions to modify and maintain behavior rather than experiential processes that focus on changing thoughts, emotions, and awareness of behavior. As previously mentioned, the process of change in the TTM can be categorized into experiential and behavioral aspects, with the latter having a stronger association with physical activity [39]. Among the facilitators in this study, behavioral aspects can be classified into the following categories: reward, opportunity, relationship, record, digital device, and time. Without efforts to develop exercise habits, facilitators are likely to remain unrecognizable. All categories in the heat map of facilitators had few mentions of precontemplation. In contrast, mentions of facilitators increased during the behavior change stage following the contemplation stage, suggesting that respondents can write about the facilitators based on their own experiences. An interview study examining the relationship between behavior change stages and influencing factors reported more statements about barriers and fewer about the benefits of exercise during precontemplation [40]. A telephone study of middle-aged Australian women reported that people in the precontemplation period lacked knowledge and family support [41]. Based on these results, interventions during the precontemplation stage should focus on providing knowledge and increasing awareness.

Applications and digital devices were mentioned as facilitating factors. Digital health interventions are becoming increasingly prevalent in exercise routines and positively associated with increased physical activity [42]. Mechanisms are being established to acquire user data through digital devices and to further encourage behavior change [38]. Text mining is actively used in this context. For example, text-mining analysis of messages from sexual and reproductive health information services in Kenya revealed distinct patterns of interest based on sex and age [43]. This study provides valuable insights into tailoring information dissemination based on the attributes of the target audience. Further studies are required to clarify the mechanisms of behavior change through digital devices.

### Strengths and Limitations

This study has several strengths. First, it visualizes fluid variations in the inhibitors and facilitators during the process of acquiring exercise habits. Inhibitors that need to be overcome step-by-step to acquire exercise habits were identified. Second, this study offers a new perspective for evaluating effective exercise interventions. The findings of this study can be used to develop intervention strategies based on inhibitors and facilitators. A meta-analysis of intervention studies on exercise habit formation found that interventions tailored to an individual’s stage of behavior change were not necessarily more effective than those that were not [43]. Furthermore, interventions focusing on self-efficacy are more effective than those based solely on behavior change stages. Interventions should consider the stage of change and factors, such as decisional balance, self-efficacy, and the process of change [44]. Third, text mining was used to clarify the behavior change process. A text-mining study analyzing free-text descriptions of exercise motivation among college students found that the high-motivation group referred to enjoyment, sports, and participation, whereas the low-motivation group mentioned dislike, rivals, and partners [21]. A previous study on coal miners applied latent Dirichlet allocation with a topic model to identify key factors, including physical and psychosocial environments, individual characteristics, and health beliefs [22]. Unlike previous studies that either focused on comparing subgroups (high vs low motivation) or identifying general themes using topic modeling, our study integrated multiple analytical approaches to comprehensively examine the dynamics of behavior change. Specifically, we applied correspondence analysis to visualize fluid transitions in frequently mentioned words across the stages of change, enabling the identification of stage-specific language patterns. Furthermore, we interpreted the extracted terms and inductively developed conceptual categories, which were then linked with participants’ behavioral stages through heat maps. This multifaceted approach allowed for a deeper understanding of how inhibitors and facilitators evolve during the process of acquiring exercise habits, contributing new insights to the theoretical and practical development of stage-based interventions.

This study has several limitations. First, due to the cross-sectional design of this study, causal relationships between behavior change stages and the reported inhibitors or facilitators cannot be inferred. To establish temporal sequences and better understand the dynamics of exercise behavior change, future longitudinal studies are warranted. Second, researchers’ subjectivity may have influenced the results, particularly in the process of categorizing inhibitors and facilitators and developing coding rules. However, to minimize this influence, we enhanced the validity of the categorization by having 2 researchers independently review the responses that included all frequently used words. Third, because this study was conducted among individuals registered with an internet panel, the generalizability of the findings may be limited. To assess potential biases, we compared the distribution of household income and behavior change stages in our sample with those reported in national datasets. Compared with the general Japanese population, respondents in this study tended to have higher household income and were more likely to be in the preparation and subsequent stages. These results suggest that the participants in our study may represent a subgroup with higher internet literacy, greater financial resources, and a stronger interest in health behaviors. Detailed comparisons with national statistics are presented in Multimedia Appendix 8 and Multimedia Appendix 9. Fourth, all data, including the self-assessment of behavior change stages and the open-ended responses, were self-reported and therefore may be subject to recall bias or social desirability bias. Fifth, the survey was conducted during a period influenced by the COVID-19 pandemic, which may have affected respondents’ exercise behaviors, perceived barriers, and available opportunities. These external factors could have shaped both the frequency and content of the reported inhibitors and facilitators.

### Conclusions

These findings contribute to a deeper understanding of the dynamic mechanisms underlying behavior change, particularly in relation to exercise habit formation. By identifying how specific inhibitors and facilitators shift across behavior change stages, this study emphasizes that exercise promotion cannot be effectively approached through one-size-fits-all strategies. Instead, the results highlight the importance of tailoring interventions to individuals’ readiness to change, as defined by their current stage.

For health practitioners, this suggests the need for careful stage-based assessment prior to intervention, enabling the provision of timely, stage-appropriate support—for example, motivational strategies for those in early stages, and more structural or logistical support (eg, time management, opportunity creation) for those further along the change process. In addition, insights into emerging facilitators, such as the use of personal rewards and health monitoring, can inform the development of new tools and counseling techniques to support sustained engagement.

For policymakers, the findings advocate for integrating behavioral stage frameworks into the design and implementation of public health initiatives. By incorporating simple assessments of behavioral readiness into national health checkups or workplace wellness programs, interventions can be more effectively segmented and personalized. Such approaches have the potential to improve program efficiency, maximize behavioral impact, and ultimately contribute to long-term increases in population-level physical activity and health outcomes.

## Supplementary material

10.2196/77400 Multimedia Appendix 1 Frequently used word list for inhibitors with 10 or more occurrences.

10.2196/77400 Multimedia Appendix 2 Frequently used word list for facilitators with 10 or more occurrences.

10.2196/77400 Multimedia Appendix 3 Categories and behavior change stages by inhibitors and facilitators.

10.2196/77400 Multimedia Appendix 4 Gender-stratified distribution of inhibitor categories across behavior change stages.

10.2196/77400 Multimedia Appendix 5 Gender-stratified distribution of facilitator categories across behavior change stages.

10.2196/77400 Multimedia Appendix 6 Education-stratified distribution of inhibitor categories across behavior change stages.

10.2196/77400 Multimedia Appendix 7 Education-stratified distribution of facilitator categories across behavior change stages.

10.2196/77400 Multimedia Appendix 8 Comparison of behavior change stages distribution in this study and the national database of specific health checkups.

10.2196/77400 Multimedia Appendix 9 Comparison of household income distribution in this study and the comprehensive survey of living conditions.

10.2196/77400 Multimedia Appendix 10 The STROBE reporting checklist.

10.2196/77400 Multimedia Appendix 11 Checklist for Reporting Results of Internet E-Surveys (CHERRIES).

10.2196/77400 Checklist 1 STROBE checklist.

10.2196/77400 Checklist 2 CHERRIES checklist.

## Abbreviations

CHERRIES: Checklist for Reporting Results of Internet E-Surveys
STROBE: Strengthening the Reporting of Observational Studies in Epidemiology
TTM: transtheoretical model
