# Supplementary material for: Mapping the Dynamics of Inhibitors and Facilitators of Exercise Behavior Within the Transtheoretical Model: Nationwide Cross-Sectional Study Using Text Mining Analysis
Source: Interact J Med Res. 2025 Oct 24;14:e77400. doi: 10.2196/77400 (PMC12551974; doi:10.2196/77400)
Supplement: Multimedia Appendix 1 [file ijmr-v14-e77400-s001.docx]

# Multimedia Appendix 1. Frequently used word list for inhibitors with 10 or more occurrences

| No | Japanese | English | Occurrence |  | No | Japanese | English | Occurrence |
| --- | --- | --- | --- | --- | --- | --- | --- | --- |
| 1 | ない | nothing | 424 |  | 41 | ウォーキング | walking | 19 |
| 2 | 時間 | time | 387 |  | 42 | 場所 | location | 19 |
| 3 | ない | not | 370 |  | 43 | 身体 | physical | 19 |
| 4 | する | do | 243 |  | 44 | いる | be | 18 |
| 5 | 運動 | exercise | 209 |  | 45 | にくい | difficult | 18 |
| 6 | 仕事 | job | 154 |  | 46 | 気力 | willpower | 18 |
| 7 | 特に | especially | 139 |  | 47 | 子育て | parenting | 18 |
| 8 | ある | exist | 89 |  | 48 | ん | non | 17 |
| 9 | できる | capable | 77 |  | 49 | 好き | like | 17 |
| 10 | なる | become | 67 |  | 50 | 膝 | knee | 17 |
| 11 | 疲れる | exhausted | 58 |  | 51 | お金 | money | 16 |
| 12 | やる気 | eagerness | 49 |  | 52 | とれる | get | 16 |
| 13 | 取れる | obtain | 47 |  | 53 | 雨 | rainfall | 16 |
| 14 | めんどくさい | troublesome | 46 |  | 54 | 介護 | caregiving | 16 |
| 15 | 継続 | continuation | 45 |  | 55 | 暑い | hot | 16 |
| 16 | 忙しい | busy | 44 |  | 56 | 嫌い | dislike | 15 |
| 17 | 面倒 | hassle | 42 |  | 57 | 行く | go | 15 |
| 18 | 体力 | stamina | 40 |  | 58 | 多い | numerous | 15 |
| 19 | 家事 | chores | 36 |  | 59 | 寒い | chilly | 14 |
| 20 | 習慣 | routine | 34 |  | 60 | 疲れ | fatigue | 14 |
| 21 | 体調 | condition | 33 |  | 61 | かかる | cost | 13 |
| 22 | 天気 | weather | 33 |  | 62 | 育児 | childcare | 13 |
| 23 | 無い | missing | 33 |  | 63 | 外 | outside | 13 |
| 24 | やる | perform | 32 |  | 64 | 歩く | walk | 13 |
| 25 | 天候 | climate | 32 |  | 65 | どう | how | 12 |
| 26 | 続く | persist | 31 |  | 66 | 気温 | temperature | 12 |
| 27 | 確保 | secure | 30 |  | 67 | 苦手 | unskilled | 12 |
| 28 | モチベーション | motivation | 26 |  | 68 | 腰痛 | backache | 12 |
| 29 | 難しい | challenging | 25 |  | 69 | 続ける | maintain | 12 |
| 30 | 気持ち | feeling | 23 |  | 70 | 家 | house | 11 |
| 31 | 出来る | achievable | 23 |  | 71 | 弱い | weak | 11 |
| 32 | 面倒くさい | bothersome | 23 |  | 72 | 体 | body | 11 |
| 33 | 余裕 | capacity | 23 |  | 73 | 毎日 | daily | 11 |
| 34 | なかなか | hardly | 21 |  | 74 | ぬ | no | 10 |
| 35 | わかる | understand | 21 |  | 75 | よる | depending | 10 |
| 36 | 悪い | bad | 21 |  | 76 | 左右 | both | 10 |
| 37 | 自分 | oneself | 21 |  | 77 | 残業 | overtime | 10 |
| 38 | ジム | gymnasium | 20 |  | 78 | 取る | take | 10 |
| 39 | 思う | think | 20 |  | 79 | 追う | pursue | 10 |
| 40 | 出る | appear | 20 |  | 80 | 分かる | comprehend | 10 |
