# Supplementary material for: Mapping the Dynamics of Inhibitors and Facilitators of Exercise Behavior Within the Transtheoretical Model: Nationwide Cross-Sectional Study Using Text Mining Analysis
Source: Interact J Med Res. 2025 Oct 24;14:e77400. doi: 10.2196/77400 (PMC12551974; doi:10.2196/77400)
Supplement: Multimedia Appendix 2 [file ijmr-v14-e77400-s002.docx]

# Multimedia Appendix 2. Frequently used word list for facilitators with 10 or more occurrences

| No | Japanese | English | Occurrence |  | No | Japanese | English | Occurrence |
| --- | --- | --- | --- | --- | --- | --- | --- | --- |
| 1 | ない | nothing | 542 |  | 41 | ウォーキング | walking | 15 |
| 2 | 特に | especially | 310 |  | 42 | 記録 | record | 15 |
| 3 | する | do | 260 |  | 43 | 効果 | effect | 15 |
| 4 | ない | not | 146 |  | 44 | 通う | attend | 15 |
| 5 | 運動 | exercise | 138 |  | 45 | 良い | good | 15 |
| 6 | ある | exist | 82 |  | 46 | スポーツ | sport | 14 |
| 7 | できる | capable | 81 |  | 47 | 家族 | family | 14 |
| 8 | なる | become | 64 |  | 48 | 簡単 | easy | 14 |
| 9 | ジム | gymnasium | 56 |  | 49 | 散歩 | strolling | 14 |
| 10 | 時間 | time | 52 |  | 50 | 体 | body | 14 |
| 11 | 一緒 | together | 48 |  | 51 | とくに | particular | 13 |
| 12 | わかる | understand | 44 |  | 52 | 家 | house | 13 |
| 13 | アプリ | application | 38 |  | 53 | 決める | determine | 13 |
| 14 | 目標 | goal | 32 |  | 54 | 見る | see | 13 |
| 15 | 仲間 | peer | 30 |  | 55 | 情報 | information | 13 |
| 16 | 継続 | continuation | 29 |  | 56 | 生活 | life | 13 |
| 17 | 無い | missing | 29 |  | 57 | 行く | go | 12 |
| 18 | 人 | human | 27 |  | 58 | 動画 | movie | 12 |
| 19 | 体重 | weight | 27 |  | 59 | 歩数 | strides | 12 |
| 20 | いる | be | 26 |  | 60 | ぬ | no | 11 |
| 21 | 健康 | health | 26 |  | 61 | ストレッチ | stretching | 11 |
| 22 | 思う | think | 26 |  | 62 | 近く | near | 11 |
| 23 | 仕事 | job | 24 |  | 63 | 支援 | support | 11 |
| 24 | YOUTUBE | youtube | 23 |  | 64 | 出る | appear | 11 |
| 25 | ん | non | 23 |  | 65 | 場所 | location | 11 |
| 26 | 習慣 | routine | 22 |  | 66 | 身体 | physical | 11 |
| 27 | 歩く | walk | 22 |  | 67 | 褒美 | reward | 11 |
| 28 | ポイント | point | 21 |  | 68 | もらえる | receive | 10 |
| 29 | モチベーション | motivation | 21 |  | 69 | やすい | likely | 10 |
| 30 | 思いつく | realize | 21 |  | 70 | 環境 | environment | 10 |
| 31 | 体力 | stamina | 21 |  | 71 | 見える | visible | 10 |
| 32 | 毎日 | daily | 20 |  | 72 | 施設 | institution | 10 |
| 33 | やる | perform | 19 |  | 73 | 自宅 | at home | 10 |
| 34 | 維持 | keep | 18 |  | 74 | 続ける | maintain | 10 |
| 35 | 自分 | oneself | 18 |  |  |  |  |  |
| 36 | 楽しい | fun | 17 |  |  |  |  |  |
| 37 | 行う | try | 17 |  |  |  |  |  |
| 38 | 出来る | achievable | 17 |  |  |  |  |  |
| 39 | 分かる | comprehend | 16 |  |  |  |  |  |
| 40 | つく | get | 15 |  |  |  |  |  |
