# Supplementary material for: Mapping the Dynamics of Inhibitors and Facilitators of Exercise Behavior Within the Transtheoretical Model: Nationwide Cross-Sectional Study Using Text Mining Analysis
Source: Interact J Med Res. 2025 Oct 24;14:e77400. doi: 10.2196/77400 (PMC12551974; doi:10.2196/77400)
Supplement: Multimedia Appendix 3 [file ijmr-v14-e77400-s003.docx]

# Multimedia Appendix 3. Categories and behavior change stages by inhibitors and facilitators

|  |  | Precontemplation | | Contemplation | | Preparation | | Action | | Maintenance | |
| --- | --- | --- | --- | --- | --- | --- | --- | --- | --- | --- | --- |
|  |  | N | % | N | % | N | % | N | % | N | % |
| Inhibitor | |  |  |  |  |  |  |  |  |  |  |
|  | Motivation | 83 | 22.7 | 66 | 18.8 | 63 | 19.7 | 10 | 13.3 | 31 | 8.0 |
|  | Health | 52 | 14.2 | 43 | 12.2 | 34 | 10.6 | 7 | 9.3 | 53 | 13.7 |
|  | Weather | 5 | 1.4 | 12 | 3.4 | 21 | 6.6 | 6 | 8.0 | 54 | 13.9 |
|  | Family | 14 | 3.8 | 22 | 6.3 | 19 | 5.9 | 3 | 4.0 | 13 | 3.4 |
|  | Time | 83 | 22.7 | 119 | 33.8 | 95 | 29.7 | 21 | 28.0 | 90 | 23.2 |
|  | Opportunity | 7 | 1.9 | 12 | 3.4 | 9 | 2.8 | 8 | 10.7 | 12 | 3.1 |
|  | Working | 27 | 7.4 | 51 | 14.5 | 36 | 11.3 | 7 | 9.3 | 37 | 9.5 |
| Facilitator | |  |  |  |  |  |  |  |  |  |  |
|  | Reward | 12 | 3.3 | 17 | 4.8 | 15 | 4.7 | 4 | 5.3 | 30 | 7.7 |
|  | Subjectivity | 31 | 8.5 | 41 | 11.6 | 36 | 11.3 | 7 | 9.3 | 40 | 10.3 |
|  | Opportunity | 9 | 2.5 | 31 | 8.8 | 12 | 3.8 | 11 | 14.7 | 21 | 5.4 |
|  | Relationship | 11 | 3.0 | 32 | 9.1 | 21 | 6.6 | 1 | 1.3 | 28 | 7.2 |
|  | Health | 13 | 3.6 | 11 | 3.1 | 13 | 4.1 | 4 | 5.3 | 27 | 7.0 |
|  | Record | 12 | 3.3 | 13 | 3.7 | 21 | 6.6 | 5 | 6.7 | 27 | 7.0 |
|  | Digital device | 14 | 3.8 | 20 | 5.7 | 16 | 5.0 | 4 | 5.3 | 16 | 4.1 |
|  | Time | 13 | 3.6 | 23 | 6.5 | 9 | 2.8 | 4 | 5.3 | 19 | 4.9 |
| Total |  | 365 | 100 | 352 | 100 | 320 | 100 | 75 | 100 | 388 | 100 |
