# Supplementary material for: Mapping the Dynamics of Inhibitors and Facilitators of Exercise Behavior Within the Transtheoretical Model: Nationwide Cross-Sectional Study Using Text Mining Analysis
Source: Interact J Med Res. 2025 Oct 24;14:e77400. doi: 10.2196/77400 (PMC12551974; doi:10.2196/77400)
Supplement: Multimedia Appendix 8 [file ijmr-v14-e77400-s008.docx]

# Multimedia Appendix 8. Comparison of behavior change stages distribution in this study and the national database of specific health checkups

| This study in 2024 (limited to people aged 40-69) | | | |  |
| --- | --- | --- | --- | --- |
|  | Males |  | Females |  |
|  | N | % | N | % |
| Precontemplation | 121 | 24.7 | 127 | 25.8 |
| Contemplation | 83 | 16.9 | 138 | 28.0 |
| Preparation | 116 | 23.7 | 89 | 18.1 |
| Action | 24 | 4.9 | 16 | 3.2 |
| Maintenance | 146 | 29.8 | 123 | 24.9 |
| Total | 490 | 100 | 493 | 100 |
|  |  |  |  |  |
| National database of specific health checkups in FY2019 (limited to people aged 40-69) | | | | |
|  | Males |  | Females |  |
|  | N | % | N | % |
| Precontemplation | 3,696,770 | 28.4 | 2,275,056 | 21.8 |
| Contemplation | 4,306,268 | 33.0 | 4,021,610 | 38.5 |
| Preparation | 1,743,390 | 13.4 | 1,702,419 | 16.3 |
| Action | 1,225,696 | 9.4 | 1,032,054 | 9.9 |
| Maintenance | 2,057,954 | 15.8 | 1,420,974 | 13.6 |
| Total | 13,030,078 | 100 | 10,452,113 | 100 |
|  |  |  |  |  |
| National dataase of specific health checkups in FY2022 (limited to people aged 40-69) | | | | |
|  | Males |  | Females |  |
|  | N | % | N | % |
| Precontemplation | 3,841,279 | 28.5 | 2,242,950 | 21.0 |
| Contemplation | 4,207,152 | 31.2 | 4,015,369 | 37.7 |
| Preparation | 1,749,053 | 13.0 | 1,768,702 | 16.6 |
| Action | 1,331,904 | 9.9 | 1,133,767 | 10.6 |
| Maintenance | 2,343,335 | 17.4 | 1,499,540 | 14.1 |
| Total | 13,472,723 | 100 | 10,660,328 | 100 |

To assess the representativeness of participants in terms of behavioral change stages, we compared the distribution of responses in our study with publicly available data from the National Database of Health Insurance Claims and Specific Health Checkups of Japan (National database) in FY2019 (before COVID-19 pandemic in Japan) and FY 2022 (the newest data), which includes information from approximately 30 million individuals. Specifically, we focused on the responses to behavior-related questions from individuals aged 40 to 69 who participated in the Specific Health Checkups program. National database was available in https://www.mhlw.go.jp/stf/seisakunitsuite/bunya/0000177182.html.

Compared with national statistics, this study included a lower proportion of individuals in the precontemplation and contemplation stages and a higher proportion in the preparation and subsequent stages. These findings indicate that the study sample was somewhat biased toward individuals who had already initiated behavioral changes aimed at acquiring exercise habits.
