# Supplementary material for: Mapping the Dynamics of Inhibitors and Facilitators of Exercise Behavior Within the Transtheoretical Model: Nationwide Cross-Sectional Study Using Text Mining Analysis
Source: Interact J Med Res. 2025 Oct 24;14:e77400. doi: 10.2196/77400 (PMC12551974; doi:10.2196/77400)
Supplement: Multimedia Appendix 9 [file ijmr-v14-e77400-s009.docx]

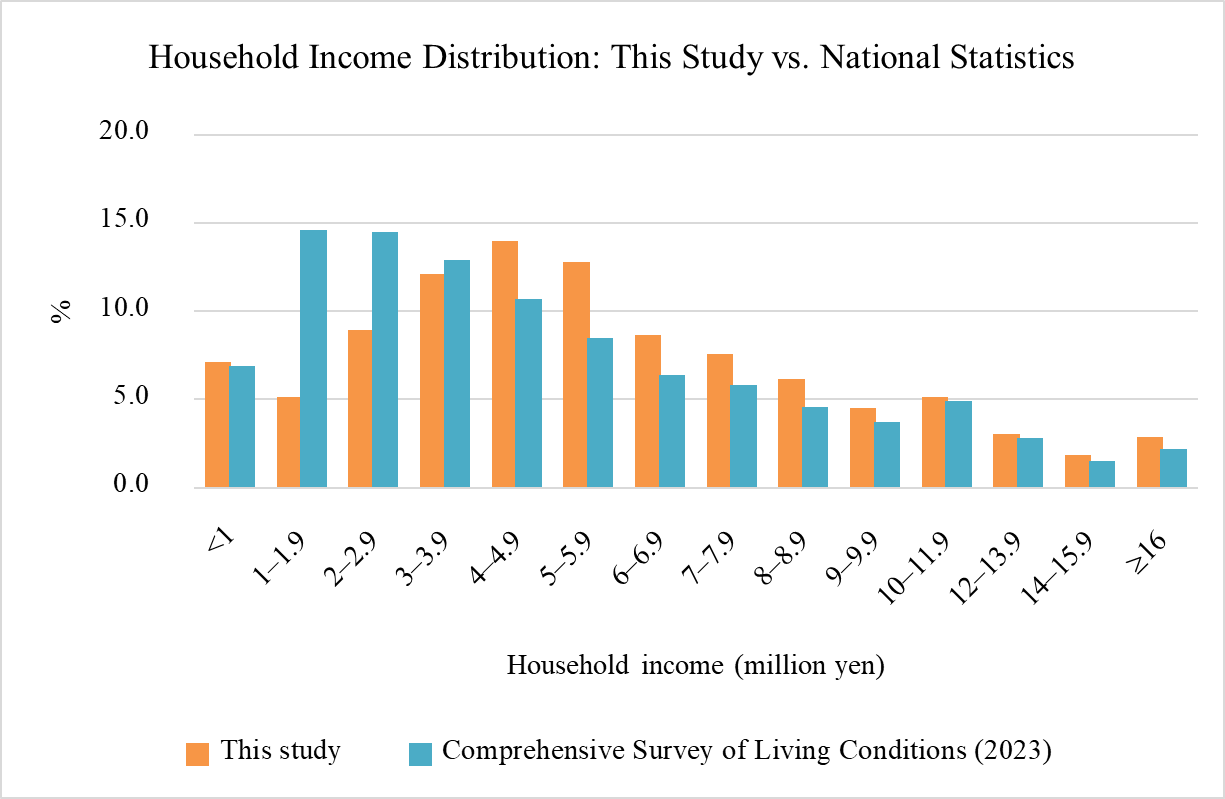


## Multimedia Appendix 9. Comparison of household income distribution in this study and the comprehensive survey of living conditions

The bar graph shows the distribution of respondents in this study compared to national statistics on household income in 2023 [1]. In this study, the respondents tended to be from households with relatively high incomes.

1. Ministry of Health, Labour and Welfare. Comprehensive Survey of Living Conditions (2023). Available from: https://www.mhlw.go.jp/toukei/saikin/hw/k-tyosa/k-tyosa23/dl/03.pdf
